# Molecular Insights into Toluene Sensing in the TodS/TodT Signal Transduction System*

**DOI:** 10.1074/jbc.M116.718841

**Published:** 2016-02-22

**Authors:** Serry Koh, Jungwon Hwang, Koushik Guchhait, Eun-Gyeong Lee, Sang-Yoon Kim, Sujin Kim, Sangmin Lee, Jeong Min Chung, Hyun Suk Jung, Sang Jun Lee, Choong-Min Ryu, Seung-Goo Lee, Tae-Kwang Oh, Ohsuk Kwon, Myung Hee Kim

**Affiliations:** From the ‡ Infection and Immunity Research Center,; the ¶ Biochemicals and Synthetic Biology Research Center, and; the ** Molecular Phytobacteriology Laboratory, Korea Research Institute of Bioscience and Biotechnology, Daejeon 305-806, Korea,; the ‖ Department of Biochemistry, College of Natural Sciences, Kangwon National University, Chuncheon, Gangwon-do 200-701, Korea, and; the § Biosystems and Bioengineering Program, University of Science and Technology, Daejeon 305-350, Korea

**Keywords:** bacterial signal transduction, biodegradation, histidine kinase, Pseudomonas, x-ray crystallography

## Abstract

TodS is a sensor kinase that responds to various monoaromatic compounds, which either cause an agonistic or antagonistic effect on phosphorylation of its cognate response regulator TodT, and controls *tod* operon expression in *Pseudomonas putida* strains. We describe a molecular sensing mechanism of TodS that is activated in response to toluene. The crystal structures of the TodS Per-Arnt-Sim (PAS) 1 sensor domain (residues 43–164) and its complex with toluene (agonist) or 1,2,4-trimethylbenzene (antagonist) show a typical β2α3β3 PAS fold structure (residues 45–149), forming a hydrophobic ligand-binding site. A signal transfer region (residues 150–163) located immediately after the canonical PAS fold may be intrinsically flexible and disordered in both apo-PAS1 and antagonist-bound forms and dramatically adapt an α-helix upon toluene binding. This structural change in the signal transfer region is proposed to result in signal transmission to activate the TodS/TodT two-component signal transduction system. Site-directed mutagenesis and β-galactosidase assays using a *P. putida* reporter strain system verified the essential residues involved in ligand sensing and signal transfer and suggest that the Phe^46^ residue acts as a ligand-specific switch.

## Introduction

As global fossil fuel consumption has increased, highly volatile aromatic hydrocarbons from petroleum by-products, such as toluene, benzene, and xylene isomers, have accumulated in the soil, water, and air. Growing public health concerns regarding prolonged exposure to these toxic substances have driven efforts to develop microbial remediation of contaminated soil and groundwater near petroleum refineries with *Pseudomonas putida* strains (1–6).

*P. putida* F1 (1, 7) and DOT-T1E (8) strains express genes encoding catabolic enzymes for the oxidation of toluene to 2-hydroxy-6-oxo-2,4-heptadienoate within the *tod* operon. TodS/TodT, a two-component signal transduction system, is responsible for regulation of *tod* operon expression in *P. putida* strains (1, 7, 8).

TodS is a member of the TodS-like family that is characterized by a unique domain architecture exclusively found in sensor kinases regulating degradation pathways (9, 10). It is an atypical histidine kinase composed of two supradomains, each containing a PAS^4^ domain and a histidine kinase (HK) domain, which are separated by a response regulator receiver (RRR) domain (10). TodS lacks a transmembrane region (7), and its N-terminal PAS domain (PAS1) binds a wide range of effectors, including toluene, to trigger increased basal autophosphorylation of TodS, leading to a phospho-signal relay to TodT and ultimately transcriptional stimulation of the *tod* operon genes by interacting with phosphorylated TodT in the P*_tod_*_X_ promoter region (11–14). A study demonstrated that the N-terminal PAS1 domain, the histidine kinase (HK1) His^190^ residue, the central RRR Asp^500^ residue, and the C-terminal histidine kinase (HK2) His^760^ residue are all required for the multistep phospho-signal to the TodT Asp^57^ residue (9). The N-terminal PAS1 domain was identified as a signal sensor, whereas the role of the C-terminal PAS domain (PAS2) in the signal transduction system remains unclear (9–11).

Another *Pseudomonas* strain, *Pseudomonas mendocina* RK1, employs the TmoS/TmoT two-component signal transduction system to control expression of the toluene-4-monooxygenase pathway (15, 16). TmoS belonging to the TodS family binds to various aromatic compounds as agonists or antagonists to regulate TmoS autophosphorylation, further demonstrating the common functional features of this protein family (16).

PAS domains have been identified in many proteins originating from archaea, bacteria, plants, and animals with highly divergent nucleotide sequences and ligand-binding capacities (17–19). As part of sensor kinases, PAS domains are often found at the N terminus, either in tandem or spaced in signal sensory modules (17, 20, 21). The functional roles of PAS domains include protein-protein interactions as well as ligand binding (17, 21, 22). PAS domains bind to a broad spectrum of ligands, such as light, oxygen, proton, heme, FMN, FAD, citrate, malate, and divalent metal cation (17, 18). In most cases, PAS functions either in a homodimer or a heterodimer with a parallel orientation, and a single ligand binds to each PAS domain (22–25).

Despite its importance in bioremediation, the structure of TodS has not yet been determined. A model of the TodS PAS1 structure predicted a hydrophobic ligand-binding pocket and suggested that four amino acid residues (Phe^46^, Ile^74^, Phe^79^, and Ile^114^) are involved in ligand binding (10). F46A, I74A, and I114A mutant variants of TodS showed significantly lower *in vitro* ligand-binding affinities, including for both agonists and antagonists, than that of wild-type (WT) TodS (10). Particularly, the F79A mutant did not respond to even high doses of ligands in both *in vitro* and *in vivo* assays, implying that the Phe^79^ residue of PAS1 is the most critical residue for ligand binding and autophosphorylation of TodS (10). Although the model structure of PAS1 is useful for predicting the ligand-binding residues, the key question of how the N-terminal PAS1 ligand-sensing domain transmits signals to the C-terminal TodS remains to be answered.

In the present study, we determined the crystal structures of the TodS PAS1 domain in complex with either toluene (an agonist) or 1,2,4-trimethylbenzene (1,2,4-TMB; an antagonist), as well that of apo-PAS1. On the basis of this structural information, we propose a mechanism for TodS molecular sensing and signal transfer to TodT using combined biochemical and *in vivo* physiological analyses.

## Experimental Procedures

### 

#### 

##### Bacterial Strains and Plasmids

The bacterial strains used and plasmids constructed in this study are listed in Table 1. Briefly, the *tod*S genes encoding TodS (residues 43–978) and dimerization helix-containing TodS (residues 23–978) were PCR-amplified using *P. putida* F1 genomic DNA as a template. The amplified TodS (residues 43–978) gene was subcloned into the NcoI and XhoI sites of pET28b(+) (Novagen), a C-terminal His-tagged protein expression vector. The TodS (residues 23–978) gene was subcloned into the BamHI and XhoI sites of the pGST-Parallel1 vector (26), an N-terminal GST-fused protein expression vector. The DNA fragments encoding PAS1 (residues 43–168), dimerization helix-containing PAS1 (residues 23–168), and PAS2 (residues 611–729) were PCR-amplified using the TodS expression plasmid and subcloned into the NcoI and XhoI sites of the pHis-Parallel1 vector (26), an N-terminal His_6_-tagged protein expression vector containing an recombinant tobacco etch virus protease cleavage site. The *tod*ST promoter and the *tod*ST gene region in *P. putida* F1 were PCR-amplified using TodSTpp-u and TodSTpp-m primers and TodST-F and TodST-R primers, respectively (Table 2). The *tod*ST promoter region was cloned into the NsiI and XbaI sites of the pBBRBB-eGFP vector (27), resulting in pBBR-P*_tod_*_ST_. Subsequently, *tod*ST was subcloned into the SacI and HindIII sites of pBBR-P*_tod_*_ST_ to express *tod*ST under the native *tod*ST promoter (pBBR-P*_tod_*_ST_-TodST) for *in vivo* assays. No green fluorescent protein gene is present in the resultant expression plasmid. The mutant variants were generated by site-directed mutagenesis using a QuikChange site-directed mutagenesis kit, according to the manufacturer's instructions (Stratagene). The L71M mutation was introduced into PAS1 (residues 43–168) to facilitate selenomethionine (SeMet) incorporation for solving the crystal structure. All introduced mutations were confirmed by DNA sequencing (Macrogen, Seoul, Korea). All primers used to create plasmids and various mutant variants are listed in Table 2.

**TABLE 1:** **Strains and plasmids used in this study**

Strain/plasmid	Relevant genotype/phenotype/characteristics	Source
**Strain**		
*E. coli*		
DH5α	^−^ Φ*80lacZ*Δ*M15* Δ*(lacZYA-argF) U169 recA1 endA1 hsdR17 (r_K_*^−^, *m_K_*^+^) *phoA supE44* λ− *thi-1 gyrA96 relA1*	Invitrogen
C41(DE3)	F^−^ *ompT hsd*S_B_ (r_B_^−^, m_B_^−^) *gal dcm* (DE3)	Lucigen
BL21 Star(DE3)	F^−^ *ompT hsd*S_B_ (r_B_^−^, m_B_^−^) *gal dcm rne*131 (DE3)	Novagen
BL21-CodonPlus (DE3)-RIPL	F^−^ *ompT hsdS* (r_B_^−^ m_B_^−^) *dcm+* Tet^R^ gal λ (DE3) *endA* Hte [*argU proL* Cam^R^] [*argU ileY leuW* Sm/Spec^R^]	Agilent
B834(DE3)	F^−^ *ompT hsdSB* (r_B_^−^m_B_^−^) *gal dcm met* (DE3)	Novagen
*P. putida*		
KT2440	Wild-type strain, spontaneous restriction-deficient of strain mt-2 cured of the TOL plasmid pWW0	Ref. 28
KT2440-PXZ	Derivative of KT2440, Δ*mexC*: P*_tod_*_X_-*lacZ*::Sm^R^	This study

**Plasmid**		
pHis-Parallel1	Amp^R^, a derivative of pFastBac-HTa(NdeI) with a polylinker of pET22B, AF097413	Ref. 26
pGST-Parallel1	Amp^R^, a derivative of pFastBac-HTa(ClaI) with a polylinker of pGEX4T1, AF097411	Ref. 26
pET28b(+)	Km^R^, *E. coli* expression vector	Novagen
TodS(23–978)	Amp^R^, wild-type TodS(23–978, BamHI/XhoI) in pGST-Parallel1	This study
TodS(43–978)	Km^R^, wild-type TodS(43–978, NcoI/XhoI) in pET-28b(+)	This study
PAS1(23–168)	Amp^R^, PAS1(23–168, NcoI/XhoI) in pHis-Parallel1	This study
F46A-PAS1(23–168)	Amp^R^, PAS1(23–168, NcoI/XhoI, F46A) in pHis-Parallel1	This study
V59A-PAS1(23–168)	Amp^R^, PAS1(23–168, NcoI/XhoI, V59A) in pHis-Parallel1	This study
F79Y-PAS1(23–168)	Amp^R^, PAS1(23–168, NcoI/XhoI, F79Y) in pHis-Parallel1	This study
W85H-PAS1(23–168)	Amp^R^, PAS1(23–168, NcoI/XhoI, W85H) in pHis-Parallel1	This study
I114V-PAS1(23–168)	Amp^R^, PAS1(23–168, NcoI/XhoI, I114V) in pHis-Parallel1	This study
E146A-PAS1(23–168)	Amp^R^, PAS1(23–168, NcoI/XhoI, E146A) in pHis-Parallel1	This study
PAS1(43–168)	Amp^R^, PAS(43–168, NcoI/XhoI) in pHis-Parallel1	This study
PAS2(611–729)	Amp^R^, PAS(611–729, NcoI/XhoI) in pHis-Parallel1	This study
PAS1(L71M)	Amp^R^, PAS(43–168, NcoI/XhoI, L71M) in pHis-Parallel1	This study
pEXT21	Sm^R^, *E. coli* expression vector	Ref. 29
pEXT20-*tod*ST-P*_tod_*_X_	Amp^R^, a pEXT20 vector containing 3,570 bp of *todST* gene and P*_tod_*_X_-*lacZ* cassette	This study
pSKY123	pBR322 vector containing 2,057-bp fragment of EcoRI and SphI fragment of the Sm^R^ gene encompassed by *mexC* gene	This study
pSKY124	pSYK123 vector containing 3,465-bp HindIII fragment of the P*_tod_*_X_-*lacZ*	This study
pSKY135	pK18mobsacB vector containing 5,528-bp fragment of the *mexC*-P*_tod_*_X_-*lacZ*-Sm-*mexC*	This study
pBR322	Amp^R^, Tet^R^, *E. coli* cloning vector	New England Biolabs, Inc.
pK18mobsacB	ATCC® 87097^TM^, Km^R^, *sacB*, *oriT*, mobilizable vector	Ref. 30
pBBRBB-eGFP	Km^R^, RK2 origin	Ref. 27
pBBR-P*_tod_*_ST_	Km^R^, a pBBRBB vector containing 504 bp of *P. putida* F1 *todST* promoter	This study
pBBR-P*_tod_*_ST_-TodST	Km^R^, a pBBR-P*_tod_*_ST_ vector containing TodST	This study
pBBR-P*_tod_*_ST_-TodST(F46A)	Km^R^, a pBBR-P*_tod_*_ST_ vector containing TodST(F46A)	This study
pBBR-P*_tod_*_ST_-TodST(V59A)	Km^R^, a pBBR-P*_tod_*_ST_ vector containing TodST(V59A)	This study
pBBR-P*_tod_*_ST_-TodST(A63V)	Km^R^, a pBBR-P*_tod_*_ST_ vector containing TodST(A63V)	This study
pBBR-P*_tod_*_ST_-TodST(F79Y)	Km^R^, a pBBR-P*_tod_*_ST_ vector containing TodST(F79Y)	This study
pBBR-P*_tod_*_ST_-TodST(W84V)	Km^R^, a pBBR-P*_tod_*_ST_ vector containing TodST(W84V)	This study
pBBR-P*_tod_*_ST_-TodST(W85R)	Km^R^, a pBBR-P*_tod_*_ST_ vector containing TodST(W85R)	This study
pBBR-P*_tod_*_ST_-TodST(W85H)	Km^R^, a pBBR-P*_tod_*_ST_ vector containing TodST(W85H)	This study
pBBR-P*_tod_*_ST_-TodST(I114V)	Km^R^, a pBBR-P*_tod_*_ST_ vector containing TodST (I114V)	This study
pBBR-P*_tod_*_ST_-TodST(V126A)	Km^R^, a pBBR-P*_tod_*_ST_ vector containing TodST (V126A)	This study
pBBR-P*_tod_*_ST_-TodST(F128L)	Km^R^, a pBBR-P*_tod_*_ST_ vector containing TodST (F128L)	This study
pBBR-P*_tod_*_ST_-TodST(A145V)	Km^R^, a pBBR-P*_tod_*_ST_ vector containing TodST (A145V)	This study
pBBR-P*_tod_*_ST_-TodST(L131D)	Km^R^, a pBBR-P*_tod_*_ST_ vector containing TodST (L131D)	This study
pBBR-P*_tod_*_ST_-TodST(V47L)	Km^R^, a pBBR-P*_tod_*_ST_ vector containing TodST (V47L)	This study
pBBR-P*_tod_*_ST_-TodST(L49D)	Km^R^, a pBBR-P*_tod_*_ST_ vector containing TodST (L49D)	This study
pBBR-P*_tod_*_ST_-TodST(E58A)	Km^R^, a pBBR-P*_tod_*_ST_ vector containing TodST (E58A)	This study
pBBR-P*_tod_*_ST_-TodST(E146A)	Km^R^, a pBBR-P*_tod_*_ST_ vector containing TodST (E146A)	This study
pBBR-P*_tod_*_ST_-TodST(E146L)	Km^R^, a pBBR-P*_tod_*_ST_ vector containing TodST (E146L)	This study
pBBR-P*_tod_*_ST_-TodST(R148A)	Km^R^, a pBBR-P*_tod_*_ST_ vector containing TodST (R148A)	This study
pBBR-P*_tod_*_ST_-TodST(R148M)	Km^R^, a pBBR-P*_tod_*_ST_ vector containing TodST (R148M)	This study
pBBR-P*_tod_*_ST_-TodST(Y691I)	Km^R^, a pBBR-P*_tod_*_ST_ vector containing TodST (Y691I)	This study
pBBR-P*_tod_*_ST_-TodST(A703S)	Km^R^, a pBBR-P*_tod_*_ST_ vector containing TodST (A703S)	This study
pBBR-P*_tod_*_ST_-TodST(Y619A/E620A)	Km^R^, a pBBR-P*_tod_*_ST_ vector containing TodST (Y619A/E620A)	This study
pBBR-P*_tod_*_ST_-TodST(Δ617–623)	Km^R^, a pBBR-P*_tod_*_ST_ vector containing TodST (Δ617–623)	This study
pBBR-P*_tod_*_ST_-TodST(E666A)	Km^R^, a pBBR-P*_tod_*_ST_ vector containing TodST (E666A)	This study
pBBR-P*_tod_*_ST_-TodST(L674A)	Km^R^, a pBBR-P*_tod_*_ST_ vector containing TodST (L674A)	This study

**TABLE 2:** **List of primers used for cloning and site-directed mutagenesis in this study**

Primer	Sequence (5′–3′)	Length
		*bp*
TodST-F	AAA GGA TCC AGG AGA CAT ATG AGC TCC TTG GAT A	34
TodST-R	TAT AAG CTT CTA TTC CAG GCT ATC CTT	27
TodSTpp-u	TAT ATG CAT CTC GAG AAA CGA GCC CAG TAC	30
TodSTpp-m	CCC TCT AGA AGC TTG CTA TTA CCT CTC TTC CAC C	34
mexCn-u	AAA GAA TTC GCA AGA CAG GTT CGA TAA GGG TG	32
mexCn-d	AAA GGA TCC AAT GCG CCC GGA GAT CGG	27
mexCc-u	AAA GGA TCC GAT ATC CAG CAA CTC GAC CCG	30
mexCc-d	TTT GCA TGC GTT TCT GCG CAG GCG CAA CG	29
Sm-N	AAA GGA TCC AAG CTT GAA CCT TGA CCG AAC GCA	33
Sm-C	ATA GGA TCC TTA TTT GCC GAC TAC CTT GGT GAT C	34
PAS1(23) NcoI-F	CA ACC ATG GGA AAG GAG AAA GGA TCT GAA G	30
PAS1(43) NcoI-F	CA ACC ATG GCG CTC TAC GAG TTT GTG	26
PAS1(168) XhoI-R	CAA CTC GAG TCA CTC CAA TTC CTG GTT CTT C	31
TodS(23) BamHI-F	C AAG GAT CCG ATG AAG GAG AAA GGA TCT G	29
TodS(978) XhoI-R	CCG CTC GAG TGT GCC GGA GCC CTG TCT GG	29
L71M-F	TG GAG GGG GGC GGG ATT ACT ATG GAA GAA ATA CGA GGG AAG	41
L71M-R	CTT CCC TCG TAT TTC TTC CAT AGT AAT CCC GCC CCC CTC CA	41
F46A-F2	GAT GGG CTC TAC GAG GCC GTG GGC CTT CTT GAT G	34
F46A-R2	C ATC AAG AAG GCC CAC GGC CTC GTA GAG CCC ATC	34
V59A-F	CT CAT GGA AAT GTG CTT GAA GCG AAC CAG GTC GCA TTG GAG GG	43
V59A-R	CC CTC CAA TGC GAC CTG GTT CGC TTC AAG CAC ATT TCC ATG AG	43
A63V-F	G CTT GAA GTG AAC CAG GTC GTA TTG GAG GGG GGC GGG ATT A	41
A63V-R	T AAT CCC GCC CCC CTC CAA TAC GAC CTG GTT CAC TTC AAG C	41
F79Y-F	GAA ATA CGA GGG AAG CCA TAC TGG AAG GCG CGT TGG TGG	39
F79Y-R	CCA CCA ACG CGC CTT CCA GTA TGG CTT CCC TCG TAT TTC	39
W84V-F	G AAG CCA TTC TGG AAG GCG CGT GTG TGG CAA ATT TCA AAA AAA ACC	46
W84V-R	GGT TTT TTT TGA AAT TTG CCA CAC ACG CGC CTT CCA GAA TGG CTT C	46
W85H-F	CCA TTC TGG AAG GCG CGT TGG CAT CAA ATT TCA AAA AAA ACC GAG	45
W85H-R	CTC GGT TTT TTT TGA AAT TTG ATG CCA ACG CGC CTT CCA GAA TGG	45
W85R-F	CCA TTC TGG AAG GCG CGT TGG CGT CAA ATT TCA AAA AAA ACC GAG	45
W85R-R	CTC GGT TTT TTT TGA AAT TTG ACG CCA ACG CGC CTT CCA GAA TGG	45
I114V-F	GAA TTT GTT CGC TGT GAT GTT GAG GTT CTT GGA AAA TCA GGT GGA AGA G	49
I114V-R	C TCT TCC ACC TGA TTT TCC AAG AAC CTC AAC ATC ACA GCG AAC AAA TTC	49
V126A-F	CA GGT GGA AGA GAG GTA ATA GCC GCC GAT TTT TCA TTG CTG CCA ATT TG	49
V126A-R	CA AAT TGG CAG CAA TGA AAA ATC GGC GGC TAT TAC CTC TCT TCC ACC TG	49
F128L-F	GA GAG GTA ATA GCC GTC GAT CTT TCA TTG CTG CCA ATT TGC	41
F128L-R	GCA AAT TGG CAG CAA TGA AAG ATC GAC GGC TAT TAC CTC TC	41
A145V-F	G AGC ATT GTT TAC CTT CTT GTG GAA GGG CGC AAT ATT ACC G	41
A145V-R	C GGT AAT ATT GCG CCC TTC CAC AAG AAG GTA AAC AAT GCT C	41
L131D-F	ATA GCC GTC GAT TTT TCA TTG GAT CCA ATT TGC AAT GAA GAA GGG	45
L131D-R	CCC TTC TTC ATT GCA AAT TGG ATC CAA TGA AAA ATC GAC GGC TAT	45
V47L-F	GAT GGG CTC TAC GAG TTT CTG GGC CTT CTT GAT GCT C	40
V47L-R	G AGC ATC AAG AAG GCC CAG AAA CTC GTA GAG CCC ATC	40
L49D-F	GG CTC TAC GAG TTT GTG GGC GAT CTT GAT GCT CAT GGA AAT G	42
L49D-R	C ATT TCC ATG AGC ATC AAG ATC GCC CAC AAA CTC GTA GAG CC	42
E58A-F	GCT CAT GGA AAT GTG CTT GCA GTG AAC CAG GTC GCA TTG	42
E58A-R	CAA TGC GAC CTG GTT CAC TGC AAG CAC ATT TCC ATG AGC	42
E146A-F	C ATT GTT TAC CTT CTT GCG GCA GGG CGC AAT ATT ACC GAT AA	42
E146A-R	TT ATC GGT AAT ATT GCG CCC TGC CGC AAG AAG GTA AAC AAT G	42
E146L-F	C ATT GTT TAC CTT CTT GCG CTG GGG CGC AAT ATT ACC GAT AA	42
E146L-R	TT ATC GGT AAT ATT GCG CCC CAG CGC AAG AAG GTA AAC AATG	42
R148A-F	C CTT CTT GCG GAA GGG GCA AAT ATT ACC GAT AAG AAG	37
R148A-R	CTT CTT ATC GGT AAT ATT TGC CCC TTC CGC AAG AAG G	37
R148M-F	C CTT CTT GCG GAA GGG ATG AAT ATT ACC GAT AAG AAG	37
R148M-R	CTT CTT ATC GGT AAT ATT CAT CCC TTC CGC AAG AAG G	37
Y619A/E620A-F	GCG CGT TGG AAA GCA GTG GCC GCC AAC TCT GCG GCC GGT ATT G	44
Y619A/E620A-R	C AAT ACC GGC CGC AGA GTT GGC GGC CAC TGC TTT CCA ACG CGC	43
Δ617–623-F	CCG CCT CGG AAG CGC GTT GGA AAG CCG GTA TTG TAC TGA CCG ACC	45
Δ617–623-R	GGT CGG TCA GTA CAA TAC CGG CTT TCC AAC GCG CTT CCG AGG CGG	45
E666A-F	CTG ACT CCA TCT GAC GCA AGC CCA CAG ATA AAG C	34
E666A-R	G CTT TAT CTG TGG GCT TGC GTC AGA TGG AGT CAG	34
L674A-F	CAG ATA AAG CAG CGT GCA GCC AAT TTG CTT CAG	32
L674A-R	CTG AAG CAA ATT GGC TGC ACG CTG CTT TAT CTG	32
Y691I-F	C AGT GTG GAG CGC TCC ATT CTA TGC AAA AAT GGT TC	36
Y691I-R	GA ACC ATT TTT GCA TAG AAT GGA GCG CTC CAC ACT G	36
A703S-F	CT ACG ATT TGG GCC AAT TCG AGT GTC TCG CTG ATG	35
A703S-R	CAT CAG CGA GAC ACT CGA ATT GGC CCA AAT CGT AG	35

##### Construction of the P. putida Reporter Strain

To perform the β-galactosidase assay, a *P. putida* KT2440-PXZ reporter strain was generated, which contains the TodT-binding promoter P*_tod_*_X_, *lac*Z, and the streptomycin resistance gene *Sm^R^* (P*_tod_*_X_-*lac*Z-*Sm^R^*) cassette inserted into the *mexC* gene on the *P. putida* KT2440 chromosome (28). Multiple subcloning steps were performed for reporter strain construction. First, two 539- and 509-bp fragments of the *P. putida* KT2440 *mex*C gene and the *Sm^R^* gene of pEXT21 plasmid (29) were PCR-amplified with primer combinations of mexCn-u and mexCn-d, mexCc-u and mexCc-d, and Sm-N and Sm-C, respectively. These three PCR products were digested with EcoRI and BamHI, BamHI and SphI, and BamHI, respectively. They were then subcloned into the EcoRI and SphI sites of pBR322 (New England Biolabs, Inc.), resulting in pSYK123. Subsequently, the P*_tod_*_X_-*lacZ* translational fusion cassette of pEXT20-*tod*ST-P*_tod_*_X_^5^ was subcloned into the HindIII site of pSYK123 to generate pSYK124 with the P*_tod_*_X_-*lacZ-Sm^R^* cassette encompassed by the sequence of *mex*C. Finally, 5,528 bp of the *mexC*-P*_tod_*_X_-*lacZ-Sm^R^-mexC* cassette was PCR-amplified using the primers mexCn-u and mexCc-d and inserted into SmaI-digested pK18mobsacB (30), yielding pSYK135. *P. putida* KT2440 was then transformed with pSYK135 by electroporation (31). The *K_m_*^R^ and sucrose-sensitive *Pseudomonas* transformants were double-selected on Luria-Bertani (LB) medium supplemented with 50 μg/ml kanamycin and 10% sucrose. A single-crossover event in the selected transformants was further confirmed by diagnostic PCR. *P. putida* KT2440-PXZ, a reporter strain with a chromosomally integrated P*_tod_*_X_-*lac*Z-*Sm^R^* cassette generated by a second crossover round, was finally selected on LB plates supplemented with 100 μg/ml streptomycin.

##### β-Galactosidase Assay

The *P. putida* KT2440-PXZ reporter strain was transformed with pBBR-P*_tod_*_ST_-TodST or its TodS mutants by electroporation. The transformant strains were grown overnight and diluted in fresh LB medium supplemented with kanamycin (50 μg/ml). The strains were then grown at 30 °C at 500 rpm for 5 h in a gas phase saturated with a ligand supplied over a range of 10–400 μm. β-Galactosidase activity by TodST or its TodS mutants in response to each ligand at different concentrations was measured using 4-methylumbelliferyl β-galactopyranoside (MUG; Sigma). MUG units were then calculated, as described previously (32–34).

##### Expression and Purification of Proteins

The N-terminally His_6_-tagged PAS1(43–168) and PAS2 proteins were expressed by induction with 0.5 mm isopropyl β-d-thiogalactopyranoside in the *Escherichia coli* BL21 Star (DE3) system (Invitrogen) at 18 °C overnight. All purification steps were performed with buffer A containing 50 mm Tris-HCl (pH 8.0) and 300 mm NaCl. Cultured cells were harvested, resuspended in buffer A, and ultrasonicated. The crude extracts were centrifuged at 16,000 × *g* at 4 °C for 1 h. The cell lysate was then loaded onto an Ni-NTA (Qiagen) affinity column, and the His_6_-tagged proteins were eluted with 200 mm imidazole. The eluted proteins were further purified by recombinant tobacco etch virus protease treatment, size exclusion chromatography (SEC) using a Superdex G75 column (GE Healthcare), and additional Ni-NTA affinity chromatography to remove His_6_ tags and uncut His_6_-tagged proteins. The purified proteins in buffer A were concentrated to 15 mg/ml and stored at −80 °C for use. N-terminally His_6_-tagged PAS1(23–168) and its mutant proteins were expressed in the *E. coli* C41(DE3) system (Lucigen) using the same method as for PAS1(43–168) and were purified in buffer A, supplemented with 5% glycerol and 2 mm DTT, using only Ni-NTA affinity chromatography for use in the isothermal titration calorimetry (ITC) experiments. SeMet-substituted PAS1 (L71M) was expressed in *E. coli* B834 (DE3) (Novagen), a methionine auxotroph strain, in minimal medium containing 50 mg/ml SeMet. The purification procedure for SeMet-PAS1 (L71M) was identical to that for the native protein except for the addition of 5 mm methionine to all of the buffers. The C-terminal His_6_-tagged TodS(43–978) protein was expressed in the *E. coli* BL21 Star (DE3) system (Invitrogen) and purified by the same procedure as for PAS1(43–168), using only Ni-NTA affinity chromatography and SEC with a Superdex G200 column (GE Healthcare). The N-terminally GST-fused TodS(23–978) protein was expressed in *E. coli* BL21-CodonPlus (DE3)-RIPL system (Agilent) as described above. Cultured cells were harvested; resuspended in phosphate-buffered saline (PBS) (pH 7.3; LPS Solution, Daejeon, Korea), 5% glycerol, 2 mm DTT, and protease inhibitor mixture (GenDEPOT); and ultrasonicated. The crude extracts were centrifuged at 16,000 × *g* at 4 °C for 1 h. The cell lysate was then loaded onto a glutathione-Sepharose 4 Fast Flow (GE Healthcare) affinity column. The column was washed with buffer containing 50 mm Tris-HCl (pH 8.0) and 300 mm NaCl, 5% glycerol, 2 mm DTT, and protease inhibitor mixture, and the GST-fused proteins were eluted with 10 mm reduced glutathione. The eluted proteins were further purified by recombinant tobacco etch virus protease treatment, SEC using a Superdex G200 column, and GST affinity chromatography to remove GST and uncut GST-fused proteins. The purified TodS(23–978) protein was used for SEC combined with multiangle light scattering (SEC-MALS) and transmission electron microscopy (TEM) analyses to determine the absolute molecular mass as well as functional dynamic structures upon ligand bindings.

##### Crystallization, Diffraction, and Structure Determination

Crystallization of PAS1(43–168) was performed by the sitting drop vapor diffusion method with commercially available sparse matrix screening kits at 21 °C. Initial crystals were grown in several different conditions, and the crystals for x-ray diffraction were optimized in conditions of 2.8–3.0 m ammonium phosphate, and 0.1 m Tris-HCl (pH 8.0–8.5) with PAS1 (5–10 mg/ml). For complex formation, PAS1 and each ligand were mixed in a 1:2 molar ratio and incubated on ice for 1 h. Crystals of PAS1 complexed with toluene and 1,2,4-TMB, respectively, were produced under the same conditions as apo-PAS1. To avoid mixing of volatile organic compounds, crystallization plates were kept in separate growth incubators. Crystals of the complexes were optimized by both hanging and sitting drop vapor diffusion methods at 21 °C. Crystals of the SeMet-substituted PAS1 (L71M) protein (10 mg/ml) and toluene complex in a 1:2 molar ratio were grown and optimized under conditions of 3.0 m ammonium phosphate and 0.1 m Tris-HCl (pH 8.0). The complex crystals were transferred to the crystallization solution functioning as a cryosolution and diffracted on a −173 °C nitrogen gas stream. Single-wavelength anomalous diffraction data for the SeMet-substituted complex was collected at a resolution of 1.65 Å at beamline 5C of the Pohang Accelerator Laboratory (Pohang, Korea). Crystals of apo-PAS1 and its complex with 1,2,4-TMB were diffracted at beamline 17A of the Photon Factory (Tsukuba, Japan), at a resolution of 1.5 and 2.0 Å, respectively. All data were processed using the HKL2000 software package (35). The structure of the SeMet-PAS1 and toluene complex was initially determined by utilizing the anomalous signals from selenium atoms with the AutoSol phasing module, and density modification and automatic model building were performed using AutoBuild from the PHENIX software package (36). The structures of apo-PAS1 and its complex with 1,2,4-TMB were solved by molecular replacement in MOLREP (37) using the SeMet-PAS1 and toluene complex structure as a template structure. All structures were revised using COOT (38) and refined with REFMAC5 (39). The refinement process included the translation-liberation-screw procedure. Crystallographic data are summarized in Table 3.

**TABLE 3:** **Data collection, phasing, and refinement statistics**

	Apo-PAS1	SeMet-PAS1-toluene	PAS1–1,2,4-TMB
**Data collection**			
Space group	P2_1_	P2_1_2_1_2_1_	P2_1_2_1_2_1_
Cell dimensions			
*a, b, c* (Å)	41.11, 49.31, 56.22	41.34, 47.74, 126.52	45.39, 51.02, 100.73
α, β, γ (degrees)	90, 109.22, 90	90, 90, 90	90, 90, 90
Wavelength	0.9795	0.9794	0.9795
Resolution (Å)	50–1.70 (1.73–1.70)*^a^*	50–1.65 (1.68–1.65)	50–1.96 (1.99–1.96)
No. of total reflections	174,101	398,442	235,138
No. of unique reflections	23,475	30,287	17,149
Redundancy	7.4 (7.3)	13.2 (12.6)	13.7 (11.9)
Completeness (%)	100.0 (100.0)	97.9 (99.5)	98.8 (98.8)
*R*_sym_ (%)*^b^*	10.2 (61.7)	9.4 (31.7)	12.0 (52.9)
*I*/σ(*I*)	32.94 (5.00)	48.62 (12.12)	49.98 (6.94)

**Refinement**			
Resolution (Å)	25.0–1.70	25.0–1.65	30.0–2.00
No. of reflections	22,211	28,907	14,675
*R*_work_/*R*_free_*^c^*	0.15/0.22	0.18/0.23	0.20/0.27
Model composition			
Protein	247 aa	249 aa	242 aa
Waters	229	308	160
Ligands		2 toluene	2 1,2,4-TMB
Root mean square deviations			
Bond lengths (Å)	0.022	0.021	0.017
Bond angles (degrees)	2.039	2.024	1.868

*^a^* The numbers in parentheses describe the relevant value for the highest resolution shell.

*^b^ R*_sym_ = Σ|I*_i_* − 〈*I*〉|/Σ*I*, where *I_i_* is the intensity of the *i*th observation and 〈*I*〉 is the mean intensity of the reflections.

*^c^ R*_work_ = Σ‖*F_o_*| − |*F_c_*‖/Σ|F*_o_*|, crystallographic *R* factor, and *R*_free_ = Σ‖*F_o_*| − |*F_c_*‖/Σ|*F_o_*|, where all reflections belong to a test set of randomly selected data.

##### ITC

The interactions between dimerization helix-containing PAS1(23–168) and ligand were analyzed by performing ITC experiments. The purified N-terminally His_6_-tagged PAS1(23–168) and its mutant proteins were dialyzed against a buffer containing 50 mm Tris-HCl (pH 7.5), 200 mm KCl, 2 mm MgCl_2_, 1 mm DTT, and 0.1 mm EDTA. These samples were then degassed by vacuum aspiration for 15 min, and ethanol was added to the samples (final concentration of 0.2%) prior to loading. Ligands containing 0.2% ethanol were diluted into the degassed buffer (10, 11). Titration was carried out at 25 °C. The calorimetric assay was performed using a VP-ITC (MicroCal Inc.). The stirring speed was 300 rpm, and the thermal power was recorded every 10 s. A concentration of 2–6 mm toluene or *m*-xylene in 0.2% ethanol in a syringe was titrated against 50 μm (calculated as a dimer) protein in the reaction cell (∼1.6 ml). Thermogram analysis of the titration was performed using the Origin package (version 7) supplied with the instrument.

##### TEM Analysis

Purified apo-TodS(23–978) and TodS(23–978) in complex either with toluene or 1,2,4-TMB were diluted to a final concentration of ∼100–200 nm with PBS (pH 7.3), 5% glycerol, 2 mm DTT, and protease inhibitor mixture. Each final solution (5 μl) was applied to a carbon-coated grid that had been glow-discharged (Harrick Plasma) for 3 min in air. The grid was immediately negatively stained using 1% uranyl acetate and examined in a Technai G2 Spirit Twin TEM (FEI) operated at 120 kV. Images were recorded on a 4k X 4k Ultrascan 895 CCD camera (Gatan). Single-particle image processing was performed using SPIDER (Health Research Inc.) as described previously (40). Averaged images were produced by alignment and classification of windowed particles from micrographs, with a total of 482 particles of apo-TodS, 411 particles of TodS in complex with toluene, and 459 particles of TodS in complex with 1,2,4-TMB. Ni-NTA gold (5 nm; Nanoprobes Inc.)-labeled TodS(43–978) proteins were visualized by negative staining as described above.

##### SEC-MALS Analysis

The absolute molecular weight of purified apo-TodS(23–978) protein in solution was determined using a Wyatt miniDAWN TREOS, a three-angle static light scattering detector (Wyatt Technology), coupled to an AKTA Purifier FPLC protein purification system equipped with a Superdex G200 column. PBS (pH 7.3) containing 10% glycerol and 5 mm DTT was filtered through a 0.20-μm filter (Toyo Roshi Kaisha Ltd., Tokyo, Japan), degassed, and used to equilibrate the Superdex G200 column at a constant rate of 0.25 ml/min at 25 °C. Calibration of the buffer solution was performed by adjusting the refractive index of 10% glycerol to 1.3448 and viscosity to 1.2125 at 25 °C. After stabilization, two reference proteins (1.5 mg/ml each), β-amylase (∼220 kDa) and bovine serum albumin (BSA; 66.4 kDa), were used to evaluate the accuracy of light scattering signals. TodS(23–978) protein (1 mg/ml) was then loaded onto the Superdex G200 column and monitored. The Rayleigh ratio was calculated based on the collected UV absorbance at 280 nm and LS2 data at 659.2 nm (refractive index), and the molecular mass was determined by Astra software version 6.0 (Wyatt Technology).

## Results

### 

#### 

##### Structure of the TodS PAS1 Sensor

**FIGURE 1.**
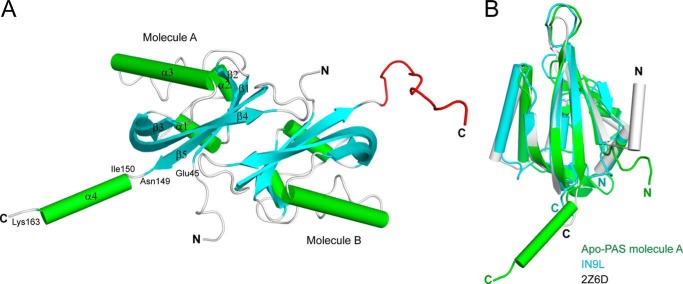
**Overall structure of apo-PAS1.** *A*, *ribbon representation* of the structure of TodS PAS1 in an asymmetric configuration. There are two PAS1 molecules in the asymmetric unit. The β-sheets and α-helices are shown in *cyan* and *green*, respectively. The C-terminal disordered region in molecule B corresponding to α4 in molecule A is displayed in *red. B*, apo-PAS1 (*green*) superimposed onto the LOV/PAS domain of the *C. reinhardtii* photoreceptor (Protein Data Bank code 1N9L; *cyan*) and the LOV1 domain of the *Arabidopsis* blue light receptor protein phototropin-2 (Protein Data Bank code 2Z6D; *gray*).

There are two PAS1 molecules in each asymmetric unit (Fig. 1*A*), resulting in an antiparallel dimer structure. The structure of each PAS1 domain is very similar to those described for other PAS domain structures, which consist of a five-stranded antiparallel β-sheet and three α-helices spanning residues 45–149 (Fig. 1*A*). An additional α-helix (α4, residues 150–163) is located immediately outside the canonical PAS fold in molecule A (Fig. 1*A*). Remarkably, the corresponding region of α4 is completely disordered in molecule B (Fig. 1*A*). The structure of the PAS1 backbone is most similar to those of the light-, oxygen-, voltage-sensitive (LOV) domain (Protein Data Bank code 1N9L, 11% sequence identity, root mean square deviation of 1.52 Å for 82 α-carbon pairs) of the *Chlamydomonas reinhardtii* photoreceptor (41) and the LOV1 domain (Protein Data Bank code 2Z6D, 11% sequence identity, root mean square deviation of 1.30 Å for 58 α-carbon pairs) of the *Arabidopsis* blue light receptor protein phototropin-2 (42), both of which bind FMN (Fig. 1*B*).

Dimerization of many PAS domains occurs mainly through an amphipathic α-helix located in the immediate upper region of the core PAS fold (42, 43). The TodS PAS1 domain constructed in this study did not include the corresponding helix region; thus, the antiparallel face-to-face dimerization observed in the TodS PAS1 structure may be an artificial consequence of the symmetry of crystal packing. In fact, secondary structure prediction suggests an α-helical region (residues 32–43) in the immediate upper region of the canonical PAS fold of TodS, which may be involved in the dimerization of PAS1 (see “Dimerization of PAS1”).

##### Signal Sensing by PAS1

**FIGURE 2.**
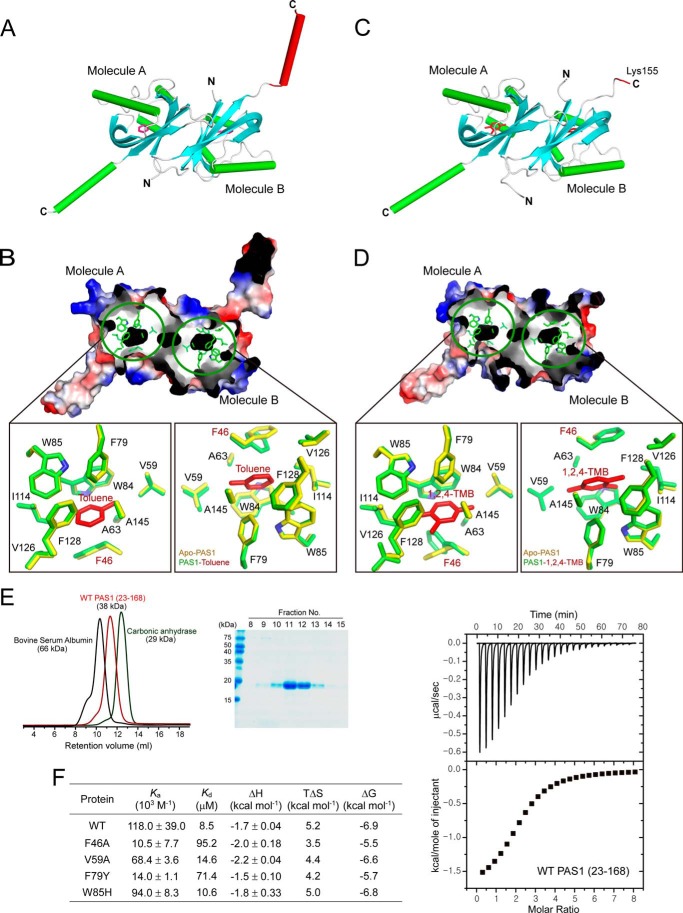
**Toluene sensing by TodS PAS1.** *A*, TodS PAS1 structure in complex with the agonist toluene. There are two toluene-bound PAS1 molecules in the asymmetric unit. Toluene molecules are shown as *red* carbon atoms. The C-terminal α-helix in molecule B corresponding to the disordered region in molecule B of apo-PAS1 is displayed in *red. B*, the toluene-binding pocket. The pocket is shown in *black* in an *electrostatic surface representation*. The detailed environment of toluene sensing by PAS1 is *magnified* for both molecules A and B. Toluene (*red*) is surrounded by hydrophobic PAS1 residues (*green*). *C*, PAS1 structure in complex with the antagonist 1,2,4-TMB. There are two antagonist-bound PAS1 molecules in the asymmetric unit. The 1,2,4-TMB molecules are shown as *red* carbon atoms. The C-terminal disordered region in molecule B corresponding to the disordered region in molecule B of apo-PAS1 is displayed in *red. D*, the 1,2,4-TMB-binding pocket. The pocket is shown in *black* in an *electrostatic surface representation*. The detailed environment of 1,2,4-TMB-binding by PAS1 is *magnified* for both molecules A and B. Notably, 1,2,4-TMB (*red*) is surrounded by the same hydrophobic residues (*green*) as those in the toluene-binding pocket. The corresponding residues (*yellow*) responsible for forming the hydrophobic pocket in apo-PAS1 are superimposed onto the toluene- and 1,2,4-TMB-binding residues, respectively. Gly^48^ is not shown in *B* and *D*, because only the side chains of all residues are displayed. *E*, SEC analysis of purified His_6_-tagged PAS1(23–168). Eluted PAS1 was compared with the molecular weight standard markers BSA (66 kDa) and carbonic anhydrase (29 kDa) (*left*) and analyzed by SDS-PAGE (*right*). *F*, thermodynamic parameters of toluene-binding to PAS1(23–168) and its mutants. The best fitting results were obtained with a “one set of binding” sites model using the ORIGIN software package (MicroCal). The heat data generated by the toluene addition to the reaction buffer were subtracted from the heat data generated from the reaction of each protein variant with toluene. The typical ITC profile for the binding of toluene molecules to WT PAS1(23–168) is displayed.

Toluene is a key signal effector required to activate the TodS/TodT signal transduction system. We determined the structure of PAS1 in complex with the agonist toluene. The overall structure of the complex is generally identical to that of apo-PAS1 (Fig. 2*A*). The agonist is positioned in a hydrophobic pocket formed by the hydrophobic residues Phe^46^, Gly^48^, Val^59^, Ala^63^, Phe^79^, Trp^84^, Trp^85^, Ile^114^, Val^126^, Phe^128^, Ala^145^, and Gly^147^ in each PAS1 molecule (Fig. 2*B*). The buried surface area of the pocket is 247.27 Å^2^, as calculated using PISA software (44). In detail, the benzene ring of toluene is placed into the area formed mainly by the aromatic amino acids Phe^46^, Phe^79^, Trp^84^, Trp^85^, and Phe^128^. Ala^63^, Ile^114^, Val^126^, and Gly^147^ are involved in the interaction as well. The methyl group of toluene is surrounded by the residues Gly^48^, Val^59^, Ala^63^, and Ala^145^.

We further determined the structure of PAS1 in complex with the antagonist 1,2,4-TMB (Fig. 2*C*). The same amino acid residues as those in the hydrophobic pocket are involved in the interaction with the antagonist (Fig. 2*D*). The only difference between PAS1 interactions with the agonist and those with the antagonist was found at the Phe^46^ position. The aromatic ring of the residue in complex with the antagonist is tilted ∼80° relative to that of Phe^46^ in complex with toluene in both molecules A and B (Fig. 2, *B* and *D*; see “Discussion” for details).

The residues critical for binding to ligand were further evaluated by ITC analysis using purified PAS1(23–168) and its mutant proteins, which exist as a dimer in solution (Fig. 2*E*). The PAS1 protein was titrated with toluene, and the ITC data were integrated and fitted into a “one set of binding sites” model, which yielded a stoichiometry of 1:1 and a binding affinity (*K_d_*, dissociation constant) of 8.5 μm for each molecule in the PAS1 dimer (Fig. 2*F*). The binding affinity of toluene was significantly reduced with mutations at positions F46A and F79Y and was mildly reduced with mutations at V59A and W85H in PAS1 (Fig. 2*F*). The binding of toluene to the mutants revealed a ∼1.5–11-fold weaker interaction than that of WT. The thermodynamic parameters of toluene binding to the proteins determined by ITC are summarized in Fig. 2*F*.

Strikingly, the structural conformation of the C-terminal region (residues 150–163), which is directly connected to the C-terminal kinase domain, differs according to which ligand is present. As described, in the apo-PAS1 structure, the helix is completely disordered in molecule B (Fig. 1*A*). In the PAS1 structure with toluene, the corresponding region is dramatically reconstructed into an α-helix (Fig. 2*A*). In the case of the antagonist 1,2,4-TMB, the equivalent region is completely disordered, and there was no electron density for amino acid residues after Lys^155^ in molecule B (Fig. 2*C*). It is important to note that molecule A in all structures exhibits an identical α-helix (α4) conformation in the C-terminal region (residues 150–163). These results imply molecular signal transduction to the C-terminal kinase domain of TodS by effector-dependent PAS1 sensing. Hereafter, we refer to the PAS1 C-terminal region (residues 150–163) as the signal transfer region (STR).

##### In Vivo Validation of the Ligand-binding Residues

**FIGURE 3.**
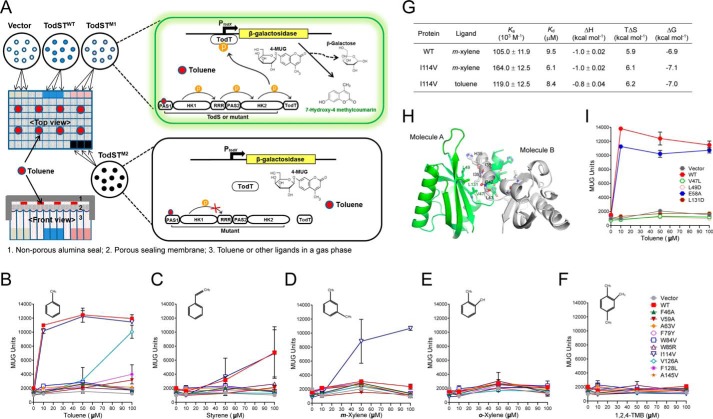
***In vivo* validation of PAS1 in the TodS/TodT signal transduction system.** *A*, schematic diagram showing the high-throughput β-galactosidase assay system. The *P. putida* KT2440-PXZ strain expressing WT-TodS/TodT or its variant mutants was grown in 96-deep well plates in the presence of a gas phase ligand in the range of 10–400 μm for β-galactosidase induction. The activity of the induced β-galactosidase was measured using the hydrolyzed fluorescent substrate, 7-hydroxy-4-methylcourmain, at 465 nm after a 1-h incubation with MUG substrate and calculated as MUG units. *B–F*, the PAS1 ligand-binding residues were evaluated for their ligand sensing and phospho-signal-relaying capacities, either upon agonist (toluene, styrene, and *m*-xylene) or antagonist (1,2,4-TMB and *o*-xylene) application in a gas phase saturated with each ligand supplied over a range of 10–100 μm. *G*, thermodynamic parameters of toluene or *m*-xylene binding to WT PAS1(23–168) and its mutant (I114V) proteins. The best fitting results were obtained with a “one set of binding sites” model using the ORIGIN software package (MicroCal). The heat data generated by the toluene or *m*-xylene addition to the reaction buffer were subtracted from the heat data generated from the reaction of each protein variant either with toluene or *m*-xylene. *H*, dimerization of PAS1. The dimeric structure of PAS1 was modeled on the structure of the *A. vinelandii* NifL LOV domain (Protein Data Bank code 2GJ3). Hydrophobic residues predicted to be involved in dimerization are displayed in *green* and *gray* in molecules A and B, respectively. *I*, the residues involved in dimerization were evaluated using the β-galactosidase assay system in a toluene environment. All results were obtained with three independent experiments. *Error bars*, S.D.

We designed the *P. putida* KT2440-PXZ reporter strain, which was constructed by fusing the promoter region P*_tod_*_X_, the transcriptional regulator TodT-binding promotor, to *lacZ* encoding β-galactosidase for further validation of the ligand-binding residues, using a high-throughput assay system (Fig. 3*A*). The reporter strain was transformed with pBBR-P*_tod_*_ST_-TodST and its variants, and their ligand-binding and multistep-signal relaying capacities were evaluated, either upon agonist (toluene, styrene, and *m*-xylene) or antagonist (1,2,4-TMB and *o*-xylene) binding. The *P. putida* KT2440-PXZ reporter strain harboring WT TodST or its mutant-expressing plasmid was grown at 30 °C at 500 rpm for 5 h in a gas phase saturated with each ligand supplied over a range of 10–100 μm. Maximum β-galactosidase activity for WT TodS was obtained in response to 10 μm toluene (Fig. 3*B*). However, other known agonists, such as styrene and *m*-xylene, were ineffective signal effectors compared with toluene in TodS/TodT signal transduction. Styrene (Fig. 3*C*) and *m*-xylene (Fig. 3*D*) did not activate TodS/TodT signaling below 100 μm and only showed maximum β-galactosidase activities at concentrations over 200 and 400 μm, respectively (data not shown). Thus, these results further indicate that toluene is the primary signal effector of the TodS/TodT signal transduction system (10, 11). No signal was transmitted with the antagonist *o*-xylene (Fig. 3*E*) or 1,2,4-TMB (Fig. 3*F*), consistent with previously reported results (10, 11).

The PAS1 hydrophobic pocket residues critical for signal sensing were evaluated in the presence or absence of agonists or antagonists. All mutations in the pocket, except for I114V, blocked signal relay from PAS1 to HK and ultimately to TodT and completely abolished enzyme activity in the toluene environment (Fig. 3*B*). The V126A mutant showed a response similar to that of the WT in the presence of 100 μm toluene (Fig. 3*B*). A similar pattern was observed with the weak agonist styrene, considering its responsiveness to PAS1. Interestingly, the I114V mutation increased sensitivity to 50 and 100 μm *m*-xylene, whereas the sensitivity of I114V mutant to styrene and toluene remained nearly the same as that of WT TodS (Fig. 3, *B* and *C*). As mentioned, *m*-xylene acts as a very weak agonist (responding at ∼400 μm) for WT TodS. We further evaluated the mutant binding with toluene and *m*-xylene using ITC analysis (Fig. 3*G*). The binding affinity of the I114V mutant with toluene was similar (calculated as *K_d_* = 8.4 μm) to that of WT PAS1 (*K_d_* = 8.5 μm) and correlated with β-galactosidase activity (Fig. 3*B*). The binding affinity (*K_d_*) of WT PAS1 with *m*-xylene was calculated to be 9.5 μm, which is comparable with that with toluene (*K_d_* = 8.5 μm). However, the binding affinity of *m*-xylene with the I114V mutant was 6.1 μm, which is 1.5-fold higher than that of the WT protein. The replacement of isoleucine with smaller side chain-containing valine may induce better fit of *m*-xylene into the ligand-binding pocket. The mildly increased binding affinity of *m*-xylene to the mutant may enhance signal transduction, suggesting that Ile^114^ plays a role not only in ligand binding but also in ligand selectivity.

##### Dimerization of PAS1

As discussed above, the dimerization helix located in the immediate upper region (residues 32–43) of the core PAS domain was removed from the PAS1 domain (residues 43–168) for crystallization and resulted in an artificial antiparallel face-to-face dimer. Thus, we modeled the dimeric structure of PAS1 based on the structure (Protein Data Bank code 2GJ3) of the *Azotobacter vinelandii* NifL LOV domain (43). Hydrophobic residues, such as Val^47^, Leu^49^, and Leu^131^, on the outside of the β-sheet in molecule A may interact with Ile^39^ and Leu^43^, His^35^ and Ile^38^, and Gly^42^, respectively, in molecule B, and are likely to be involved in dimerization and the maintenance of structural stability (Fig. 3*H*). The V47L, L49D, and L131D mutations completely abolished β-galactosidase activity in the toluene environment (Fig. 3*I*), suggesting that these residues are essential for PAS1 dimerization. By contrast, mutation of the charged residue Glu^58^ located outside of the β-sheet did not affect PAS1 dimerization (Fig. 3*I*).

##### Role of PAS2 in the TodS/TodT System

TodS comprises two modules, each containing a PAS-type sensor domain and an autokinase domain, and is thus classified as a “double sensor kinase” family member (9). As suggested by Lau *et al.* (7), the complexity of the TodS domain structure may provide the capacity for fine tuning by the two-component signal transduction system via integrating additional signals. The *tod* pathway is oxygen-dependent, and the PAS2 domain is not responsible for sensing aromatic signal molecules; therefore, they proposed that PAS2 might be able to sense oxygen (7). However, no direct evidence supporting this hypothesis has yet been reported.

**FIGURE 4.**
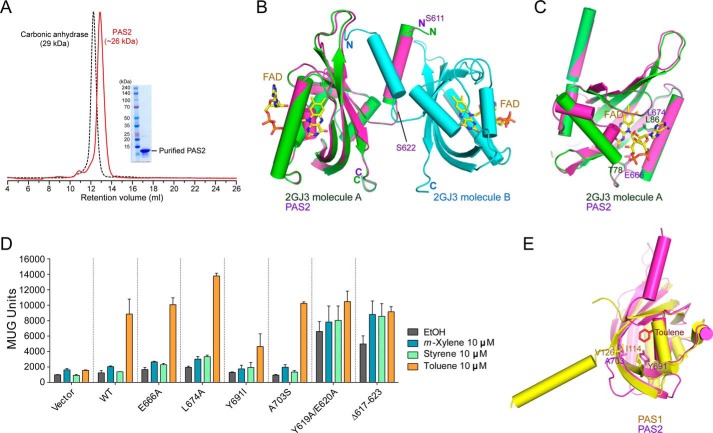
**Role of PAS2 in TodS/TodT signal transduction.** *A*, SEC analysis of purified PAS2(611–729). Eluted PAS2 protein was compared with the molecular weight standard marker carbonic anhydrase (29 kDa) and analyzed as a dimer in solution. *B*, the modeled PAS2 structure (*magenta*) based on the dimeric structure (*green* and *cyan*) of the *A. vinelandii* NifL LOV domain (Protein Data Bank code 2GJ3). FAD binding to each NifL PAS domain is shown. The N-terminal α-helix (residues Ser^611^–Ser^622^) of PAS2 predicted to be involved in dimerization is indicated. *C*, PAS2 residues Glu^666^ and Leu^674^, corresponding to the NifL FAD binding residues Thr^78^ and Leu^86^. *D*, potential PAS2 residues involved in ligand binding were assessed using the β-galactosidase assay with different ligands. The N-terminal PAS2 α-helix (residues Ser^611^–Ser^622^) predicted to be involved in dimerization, as seen in Fig. 4*B*, was also evaluated with the same assay. *E*, the modeled PAS2 was superimposed onto toluene-bound PAS1. The PAS2 residues Tyr^691^ and Ala^703^ were analyzed with the corresponding PAS1 residues Ile^114^ and Val^126^, which are responsible for toluene binding.

To better understand the phosphorelay of TodS, we attempted to crystallize PAS2 to determine the structure and uncover its function. Although it was well purified and analyzed as a dimer in solution (Fig. 4*A*), crystallization failed. The sequence analysis predicted that PAS2 may have a similar structure to that of the *A. vinelandii* NifL LOV domain (Fig. 4*B*) (Protein Data Bank code 2GJ3) (43). Thus, we modeled the structure of PAS2 (residues 611–729) using the structure of the NifL LOV domain as a template (Fig. 4*B*). Among the residues corresponding to the FAD (ligand) binding residues (Thr^78^ and Leu^86^) of NifL, Glu^666^ and Leu^674^ of PAS2 (Fig. 4*C*) were mutated to alanine, and their activity was compared with that of WT TodS in a β-galactosidase activity assay. The results showed no difference in β-galactosidase activity between the mutant variants and the WT (Fig. 4*D*). The structure of PAS1 complexed with toluene was superimposed onto the modeled structure (Fig. 4*E*). Then we tested residues Tyr^691^ and Ala^703^ in PAS2, corresponding to the critical toluene-binding residues Ile^114^ and Val^126^ in PAS1. The TodS mutants at the Tyr^691^ and Ala^703^ positions did not exhibit a toluene signal transfer capacity significantly different from that of WT TodS (Fig. 4*D*). These results are consistent with those of previous reports that the PAS2 domain does not function either as a toluene or FAD sensor (9, 10). It is worth mentioning that all results of the β-galactosidase activity assay in this study were obtained in culture medium containing limited ligand sources, in which PAS2 might not have the opportunity to bind to an unknown ligand(s). Thus, we do not rule out the possibility that PAS2 may bind to an unknown ligand(s) in order to regulate TodS/TodT signal transduction.

On the other hand, given that TodS functions as a dimer (see “SEC-MALS and TEM Analyses of TodS”), we hypothesized that dimerization of PAS2 is also important for tightly controlled phosphorelay. The modeled PAS2 structure revealed that the first α-helix surrounding residues Ser^611^–Ser^622^ (Fig. 4*B*) may be involved in PAS2 dimerization. Interestingly, deletion of the dimerization region of TodS (residues 617–623) significantly increased basal levels of β-galactosidase activity in the presence of weak agonists, such as *m*-xylene and styrene (note that these agonists at a concentration of 10 μm in a gas phase could not be sensed by WT TodS), as well as in the absence of ligand (Fig. 4*D*). The same results were obtained with TodS with a double mutation (Y619A and E620A) (Fig. 4*D*). These results indicate that maintaining the correct dimerization of PAS2 of TodS is critical for finely tuning phosphorelay to the C-terminal HK2 and TodT.

##### SEC-MALS and TEM Analyses of TodS

**FIGURE 5.**
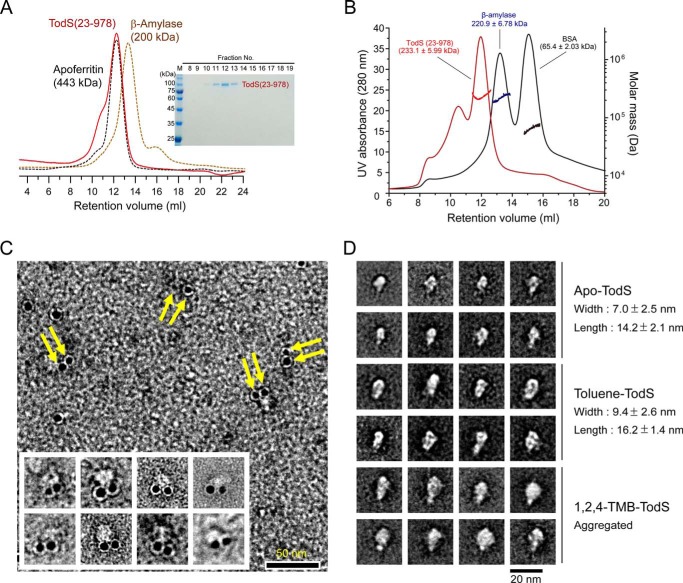
**SEC-MALS and electron microscopic analyses of TodS.** *A*, SEC analysis of purified TodS(23–978). Eluted TodS was compared with the molecular mass standard markers β-amylase (223.8 kDa) and apoferritin (443 kDa). *B*, SEC-MALS analysis of TodS(23–978). *Horizontal lines across* the *peaks* indicate the calculated molecular mass of eluted TodS(23–978) (*red*), β-amylase (*blue*), and BSA (*black*). The *inset* shows SDS-PAGE of purified TodS(23–978) used for SEC-MALS analysis. *C*, electron microscopic image of gold-labeled TodS proteins. Ni-NTA-gold labeling of C-terminal His_6_-tagged TodS(43–978) proteins was employed to define the molecular arrangement of the TodS dimer. Shown is a negative-stained image showing paired circular gold particles (*yellow arrows*), suggesting a head-to-head dimer of TodS. *Scale bar*, 50 nm. *D*, molecular appearance of TodS(23–978) proteins. Electron microscopic images of apo-TodS (*top*), TodS treated with toluene (*middle*), and TodS treated with 1,2,4-TMB (*bottom*) were visualized by negative staining. Ten representative images were selected. *Scale bar*, 20 nm.

We noticed that full-length TodS protein was unstable and that it aggregated easily. It was therefore difficult to purify and use in biochemical studies *in vitro*. The C-terminal His_6_-tagged TodS(43–978) and the N-terminal GST-fused TodS(23–978) protein were, however, relatively soluble and could be purified. SEC analysis showed that purified TodS(23–978) protein was eluted in the same fraction as the size marker protein apoferritin (443 kDa), indirectly indicating its flexible properties (Fig. 5*A*). We further employed SEC-MALS to determine the absolute molecular mass of TodS(23–978) in solution based on the angular dependence of scattered light intensity, which is independent of the molecular shapes. The retention volume (∼12 ml) of TodS(23–978) in SEC-MALS analysis was similar to the SEC result of TodS(23–978) (Fig. 5*A*); however, MALS analysis indicated that the molecular mass of this fraction was 233.1 ± 5.99 kDa (Fig. 5*B*), which corresponds to dimeric TodS(23–978). It is worth noting that aggregation of TodS(23–978) was observed during SEC-MALS analysis (Fig. 5*B*). Collectively, these results suggest that the TodS(23–978) protein exists primarily as a flexible dimeric structure in solution.

To define the molecular arrangements of TodS proteins, we performed immunogold labeling electron microscopy analysis. The neighboring position of the Ni-NTA gold particles, as shown in Fig. 5*C*, corresponding to the position of the C-terminal His_6_-tagged region of the TodS(43–978) proteins, indicated a parallel arrangement of each protein in a dimeric assembly (Fig. 5*C*). Some monomeric gold particle labeling of TodS (Fig. 5*C*) may be caused by the single focal plane of the image presented or artifactual assemblies by the TEM sample preparation procedure.

Furthermore, averaged images taken from negatively stained single particles of apo-TodS(23–978) protein provided information about the characteristics of TodS in the presence of different ligands. The apo-TodS molecule had a length of 14.2 nm and a width of 7.0 nm (Fig. 5*D*, *top*). In the structure, toluene sensing by PAS1 induced a structural change (especially residues 150–163) and affected TodS/TodT signal transduction. Thus, we assumed that the overall features of TodS might be affected by the presence of toluene. The toluene-sensing TodS proteins had a similarly shaped appearance to that of apo-TodS at the molecular level; however, they had a length and a width of 16.2 and 9.4 nm, respectively, demonstrating a structural change to a more straightened configuration (Fig. 5*D*, *middle*). The ligand-dependent conformational change of TodS was also supported by the changed overall features of TodS complexed with the antagonist 1,2,4-TMB, which showed mainly oligomerization (Fig. 5*D*, *bottom*). Thus, these results suggest that TodS undergoes ligand-dependent structural changes that are responsible for the activation of signal transduction.

## Discussion

The *P. putida tod* operon, comprising genes encoding enzymes that catabolize toluene, is tightly regulated by TodS/TodT two-component multistep-signal transduction in a ligand-dependent manner (9–11, 45). The N-terminal PAS1 domain in TodS acts as a sensor for benzene-derived hydrocarbons with high affinity, whereas the C-terminal PAS2 domain lacks a signal-sensing function (9–11, 45).

In this study, we determined the structure of the PAS1 sensor as an apoprotein and as a complex with either with toluene (an agonist) or 1,2,4-TMB (an antagonist) to explore the molecular mechanisms underlying how PAS1 senses effector molecules and delivers signals to C-terminal HK to regulate TodS/TodT signal transduction.

Unlike most PAS homologues that bind cofactors, such as FAD and FMN, the TodS PAS1 binds hydrophobic benzene-derived compounds. Accordingly, the ligand-binding pocket of PAS1 is surrounded by completely hydrophobic residues. The phototrophin-2 LOV1 domain (Fig. 1*B*), which has the structure most similar to that of PAS1, regulates light sensitivity through binding to FMN (42). The binding pocket for FMN in LOV1 is greater than that for toluene in TodS PAS1 and is surrounded by polar residues. Bulky and hydrophobic residues, including Trp^84^, Trp^85^, Phe^46^, Phe^79^, and Phe^128^, are involved in the interaction with toluene in TodS PAS1, whereas charged and polar residues, including Arg^133^, Asn^136^, Asn^164^, Glu^265^, and Glu^272^, are associated with FMN in LOV1. Such properties of the ligand-binding pocket of PAS1 are consistent with a recent study showing that TodS does not bind cofactors involved in redox sensing, including FAD, FMN, heme, NAD, NADH, NADP, and NADPH (46).

**FIGURE 6.**
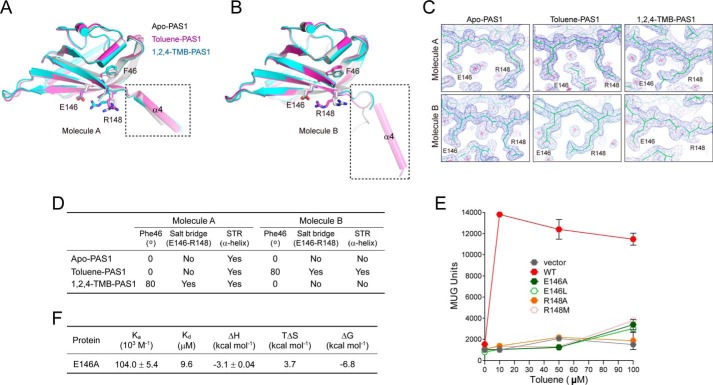
**Molecular switching by Phe^46^ for signal transfer.** *A* and *B*, toluene-bound (*magenta*) and 1,2,4-TMB-bound (*cyan*) PAS1 structures were superimposed onto the apo-PAS1 structure (*gray*). The critical residues for signal transfer (Phe^46^, Glu^146^, and Arg^148^) are displayed. The STR is indicated by the *dashed rectangular box. C*, 2*F_o_* − *F_c_* electron density maps contoured at 1.1 σ showing the Glu^146^ and Arg^148^ positions in each PAS1 structure. *D*, summary of the signal switching by Phe^46^ upon binding of different types of ligands. Position of the Phe^46^ aromatic ring in each PAS1 structure was specified as the degree of tilting relative to that of Phe^46^ in apo-PAS1. *E*, evaluation of the residues involved in signal switching with the β-galactosidase assay in a toluene environment. Results were obtained with three independent experiments. *F*, thermodynamic parameters of toluene-binding to the E146A mutant. The best fit results were obtained with a “one set of binding” sites model using the ORIGIN software package (MicroCal). The heat data generated by the toluene addition to the reaction buffer were subtracted from the heat data generated from the reaction of E146A with toluene. *Error bars*, S.E.

The structural comparison suggests that the residues ranging from 150 to 163 (STR) in PAS1 are essential for relaying phospho-signals to HK1, RRR, and HK2. The dramatic conformational change in STR from a flexible conformation to an α-helix seems to be controlled by the aromatic ring of Phe^46^ (Fig. 2, *B* and *D*). According to the type of ligand that PAS1 senses, Phe^46^ may be positioned differently to turn on or off signal transfer. In molecule A, the Phe^46^ aromatic ring is tilted ∼80° in response to the antagonist 1,2,4-TMB relative to that of Phe^46^ either in apo-PAS1 or in complex with toluene. This change led to the formation of a salt bridge between two amino acid residues, Arg^148^ and Glu^146^, on the β5 strand, whereas no such bridge was observed in either apo- or agonist-bound PAS1 (Fig. 6*A*). Nevertheless, molecule A in all structures maintains the STR region as an α4 helix (Fig. 6*A*). By contrast, in molecule B, tilting of the Phe^46^ aromatic ring occurred only in response to the agonist toluene, and this was linked to the formation of a salt bridge between Arg^148^ and Glu^146^ and the transformation of the flexible STR region (in apo-PAS1) to an α4 helix (Fig. 6*B*). Interestingly, residue Arg^148^ in molecule B of PAS1 complexed with the antagonist 1,2,4-TMB is very flexible and shows no visible electron density, whereas the electron density map of the corresponding residues in other structures is seen clearly (Fig. 6*C*). The phenomenon is probably related to the flexibility and lack of electron density in the STR region of molecule B of PAS1 complexed with 1,2,4-TMB (Figs. 2*C* and 6*B*). It is not clear whether the conformational changes are mutually affected by ligand binding to molecules A and B. The signal switching of Phe^46^ upon binding different types of ligands is summarized in Fig. 6*D*. We further assessed the importance of the Glu^146^ and Arg^148^ residues in signal transfer. In the β-galactosidase assay systems, E146A and R148A (or R148M) TodS mutations completely abolished β-galactosidase activity even in the presence of 100 μm toluene, whereas WT TodS responded to >10 μm toluene in a gas phase (Fig. 6*E*). The toluene-binding affinity of the E146A mutant dimer (*K_d_* = 9.6 μm) was very similar to that of WT PAS1 (Fig. 6*F*). Collectively, these results suggest that the Phe^46^ residue of PAS1 acts as a molecular switch to selectively control TodS/TodT signaling. Another member of the TodS-like family, TmoS, also binds to and distinguishes a wide range of aromatic carbons either as an agonist or as an antagonist (16). In fact, amino acid sequence alignments of TodS-like family sensor kinases, such as TodS, TmoS, and StyS, revealed that residues Phe^46^, Glu^146^, and Arg^148^ are strictly conserved (16). Thus, it could be hypothesized that Phe^46^ may also play a key role in the ligand sensing of TmoS. Biochemical characterizations of Phe^46^ residues in TodS-like family members should be further investigated.

**FIGURE 7.**
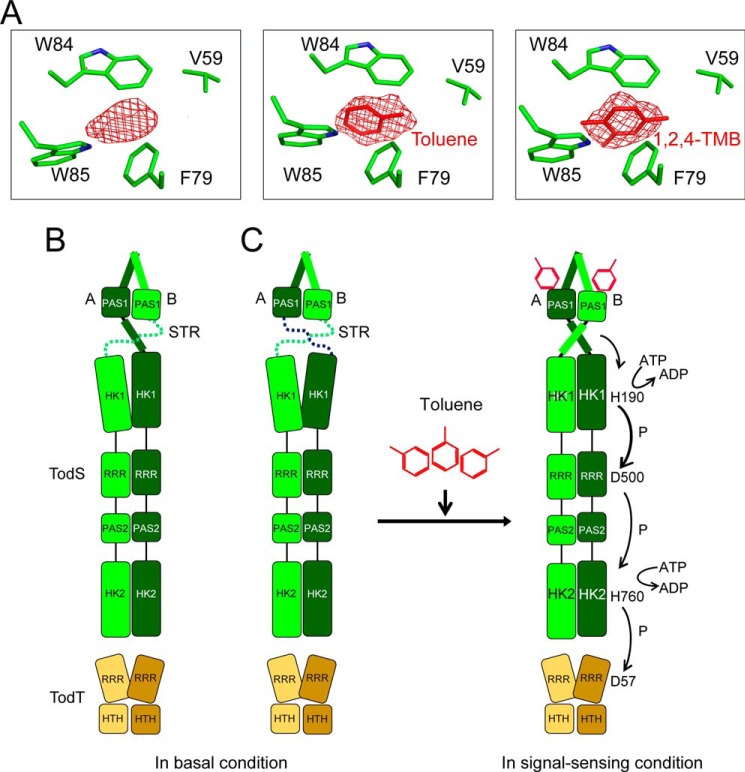
**Proposed molecular mechanism of TodS/TodT signal transduction.** *A*, the *F_o_* − *F_c_* electron density map of apo-PAS1 contoured at 3.0 σ (*left*), corresponding to the maps for toluene contoured at 3.0 σ (*center*) and 1,2,4-TMB contoured at 2.5 σ (*right*) of PAS1-ligand complexes, respectively. *B* and *C*, proposed models of TodS/TodT signal transduction. TodS exists as a dimer with a flexible nature, which might possess a basal level of autokinase activity. In this condition, the PAS1 sensor domain would not effectively deliver signals to the C-terminal HK1 via the flexible nature of STR. Thus, this conformation could not induce the functional dimeric conformation of HK1 to enable successful autophosphorylation. Upon toluene sensing at an effective level, the PAS1 STRs may be reorganized to transmit signals and induce conformational changes in TodS to align the HK1-RRR-PAS2-HK2 domains for efficient multistep phosphorelay. See “Discussion” for a full description. Molecules A and B in the PAS1 dimer are shown in *light green* and *dark green*, respectively. TodT dimers are displayed in *light* and *dark yellow*. Toluene molecules are shown in *red*.

During refinement of the apo-PAS1 structure, we noted a region of weak electron density in the hydrophobic ligand-binding pocket. The shape of the region is somewhat similar to those of benzene-derived compounds, including toluene and 1,2,4-TMB (Fig. 7*A*). It is important to note that volatile organic compounds, including benzene derivatives, are common chemical contaminants that readily evaporate to some extent in air, depending on the environmental conditions. Particularly, laboratory environments are exposed to a wide variety of chemicals and regularly contain volatile organic compounds (47), even in incubators in which protein crystals are grown. Thus, we thought that the region of weak electron density observed in the apo-PAS1 structure may have originated from contaminants in the incubator (or the laboratory environment). It may also be possible that a cellular ligand, such as an aromatic compound, was co-purified with PAS1 protein. Nonetheless, the level of contamination was probably not sufficiently high to affect PAS1-mediated TodS signaling. It is worth mentioning that PAS1 was crystallized with different ligands in separate incubators to avoid cross-contamination.

Our results showed that maintaining the correct dimerization of PAS2 is crucial to finely tune phosphorelay. PAS2 exists as a dimer in solution (Fig. 4*A*), and the region (residues 617–623) of PAS2 involved in dimerization appears to be important for tight regulation of the basal levels of β-galactosidase activity in ligand-specific TodS/TodT signal transduction (Fig. 4). The Krell group constructed a minimal form of TodS, termed Min-TodS, which contains only the N-terminal PAS1 and the C-terminal HK2 of TodS, by removal of the central HK1, RRR, and PAS2 domains (45). They demonstrated that Min-TodS binds effector molecules with affinities similar to those observed for WT TodS. They also showed that both Min-TodS and WT TodS can classify effector molecules into agonists and antagonists, indicating that the molecular determinants of effector recognition and their agonistic or antagonistic action are located in the PAS1 domain (45). However, they observed much higher basal activity of Min-TodS, whereas WT TodS was almost silent in the absence of toluene. Busch *et al.* (9) reported that TodS mutants, in which the phosphoryl-accepting amino acids His^190^, Asp^500^, and His^760^ are replaced with alanine, exhibit low basal activity that is comparable with the WT TodS system, suggesting that the low basal activity occurs irrespective of whether there is functional phosphorelay. Thus, these data suggest that all domains within TodS are required to maintain the low basal activity of the TodS system for the final accurate response of TodT because *tod* operon expression in the absence of effector molecules is useless.

Finally, taken together with the results of previous structural, biological, and *in vivo* studies, the results of TEM analysis, which revealed changes in TodS into aligned or misaligned structures, depending on the ligand employed, allowed us to propose an outline of the molecular mechanism of TodS/TodT signal transduction (Fig. 7, *B* and *C*). Structural flexibility between the sensor PAS1 and HK1 as well as the dimeric features of TodS are essential for efficient signal relay under certain environmental conditions. Toluene binding to the PAS1 sensor domain induces conformational changes in TodS that result in a functional form that is proficient for phospho-signal relay (Fig. 7, *B* and *C*). All structures of the STR of molecule A in both apo- and ligand-bound PAS1 exhibited a well structured α-helix, whereas the corresponding region in molecule B was structured differently (in all structures), depending on which ligand was employed. In the absence of a signal effector, the PAS1 sensor domain cannot provide a signal to HK1 via the flexible nature of the STR (Fig. 7*B*). This conformation cannot induce the functional dimeric conformation of HK1 to enable autophosphorylation. We cannot rule out bilateral flexibility of the STR in both molecules A and B (Fig. 7*C*). Upon toluene sensing at an effective level, the PAS1 STRs are reconstructed to induce functional reorganization of TodS and transmit signals to TodS/TodT, ultimately up-regulating the *tod* operon.

## Author Contributions

S. K. performed most of the biochemical experiments. J. H., K. G., and M. H. K. carried out the structural study. E.-G. L., S.-Y. K., and O. K. constructed the *P. putida* reporter strain. S. L., J. M. C., and H. S. J. performed EM analysis. S. K., S. J. L., C.-M. R., S.-G. L., and T.-K. O. contributed to the discussion and provided reagents. S. K., J. H., and M. H. K. designed the study and wrote the manuscript. All authors helped with data analysis.

## Footnotes

5 E.-G. Lee, S.-Y. Kim, and O. Kwon, unpublished data.

## Footnotes

4 The abbreviations used are:

PAS: Per-Arnt-Sim
HK: histidine kinase
1,2,4-TMB: 1,2,4-trimethylbenzene
STR: signal transfer region
MUG: 4-methylumbelliferyl β-galactopyranoside
RRR: response regulator receiver
SeMet: selenomethionine
Ni-NTA: nickel-nitrilotriacetic acid
SEC: size exclusion chromatography
MALS: multiangle light scattering
ITC: isothermal titration calorimetry
TEM: transmission electron microscopy
LOV: light-, oxygen-, voltage-sensitive.
